# Peripheral and central capillary non-perfusion in diabetic retinopathy: An updated overview

**DOI:** 10.3389/fmed.2023.1125062

**Published:** 2023-03-23

**Authors:** Alessio Antropoli, Alessandro Arrigo, Lamberto La Franca, Lorenzo Bianco, Eugenio Barlocci, Emanuele Fusi, Francesco Bandello, Maurizio Battaglia Parodi

**Affiliations:** Department of Ophthalmology, IRCCS San Raffaele Scientific Institute, Vita-Salute San Raffaele University, Milan, Italy

**Keywords:** peripheral capillary non-perfusion, diabetic macular ischemia, diabetic retinopathy, fluorescein angiography (FA), optical coherence tomography angiography (OCTA)

## Abstract

Capillary non-perfusion (CNP) is one of the key hallmarks of diabetic retinopathy (DR), which may develop both in the periphery and at the posterior pole. Our perspectives on CNP have extended with the introduction of optical coherence tomography angiography (OCTA) and ultra-widefield imaging, and the clinical consequences of peripheral and macular CNP have been well characterized. Fluorescein angiography (FA) continues to be the gold standard for detecting and measuring CNP, particularly when ultra-widefield imaging is available. OCTA, on the other hand, is a quicker, non-invasive approach that allows for a three-dimensional examination of CNP and may soon be regarded as an useful alternative to FA. In this review, we provide an updated scenario regarding the characteristics, clinical impact, and management of central and peripheral CNP in DR.

## Introduction

**FIGURE 1:**
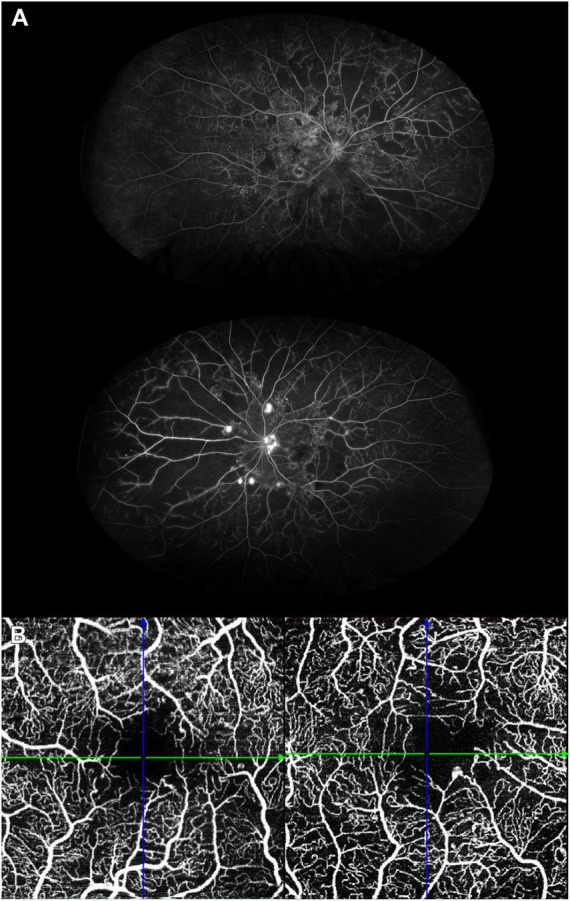
**(A)** Ultra-widefield fluorescein angiography (FA) of the right (upper image) and left (bottom image) eyes of a patient with extensive peripheral capillary non-perfusion. The right eye is in the non-proliferative stage, whereas proliferative diabetic retinopathy has developed in the left eye, which also displays the highest amount of non-perfusion areas. **(B)** Optical coherence tomography angiography of a patient with diabetic macular ischemia. The circularity of the foveal avascular zone is reduced, together with a reduction in vessel density as well.

Diabetes mellitus is a metabolic disease characterized by chronic hyperglycemia resulting from various etiological factors. As per the International Diabetes Alliance, the global prevalence of diabetes stood at 400 million in 2015 and is anticipated to escalate to 600 million by the year 2040 (1). Diabetic retinopathy (DR) is the primary cause of vision loss in elderly individuals and the most commonly occurring microvascular complication associated with diabetes mellitus. The vascular endothelium is damaged by vascular hyperglycemia through pathogenetic pathways involving advanced glycation end products, increased flux *via* the polyol pathway, activation of protein kinase C, and the generation of reactive oxygen species (2). The hyperglycemia also leads to the formation of microaneurysms and dot intraretinal hemorrhage, which are the early signs of non-proliferative diabetic retinopathy (NPDR). As the disease progresses, vasoconstriction and vascular occlusion lead to capillary non-perfusion (CNP) and ischemia, which can affect both the macular and peripheral regions (Figure 1). Finally, the last stage of DR is characterized by severe hypoxia, which causes an overexpression of vascular endothelial growth factor (VEGF), ultimately leading to proliferative diabetic retinopathy (PDR). The latter is defined by the growth of abnormal blood vessels and can be complicated by vitreous hemorrhage and retinal detachment. Additionally, widespread retinal edema caused by significant capillary leakage provokes the formation of cystoid macular edema (CME), which is the first cause of sight loss in DR (3).

In healthy eyes, the retinal capillary network that supplies the inner retinal layers is composed by four different levels of capillary plexuses: radial peripapillary capillaries, superficial capillary plexus (SCP), intermediate capillary plexus (ICP) and deep capillary plexus (DCP) (4). However, not all of them can be traced in the whole retina, since significant differences exist between specific areas: radial peripapillary capillaries are confined solely to the retinal nerve fiber layer surrounding the optic nerve head. Conversely, no plexuses are present in the rod-free central macular area, specifically the foveal avascular zone (FAZ), in order to maintain optimal visual acuity. Because of that and the high metabolic demand, the macula is very vulnerable to ischemic insults, resulting in FAZ enlargement (5).

Due to the epidemiological relevance diabetes is constantly gaining, the aim of this review is to discuss about the current knowledge regarding the pathophysiological mechanisms of CNP in DR, their consequences on the visual function and possible therapeutical repercussions on the patient management, based on the latest evidence.

## Methods

Publications in English between January 2017 and November 2022 were identified through the “advanced search” PubMed engine using the following entries: “(“non-perfusion”) AND (“diabetic retinopathy”),” yielding 87 results, and “(“diabetes”) AND (“macular”) AND [(Ischemia) OR (non-perfusion)],” resulting in 257 results. Studies published before 2017 were considered for this review if they were cited in the papers found through the aforementioned method. An individual selection of the studies found this way was carried out by the authors and those matching our scope were included in this review.

## Imaging modalities of capillary non-perfusion in diabetic retinopathy

**FIGURE 2:**
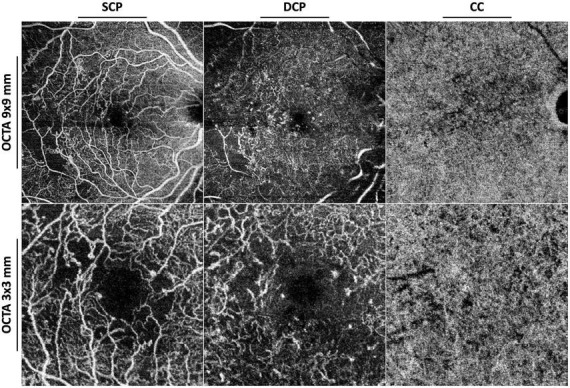
Optical coherence tomography angiography (OCTA) with 9 × 9 mm **(upper row)** and 3 × 3 mm **(lower row)** fields of view of an eye affected by proliferative diabetic retinopathy. SCP, superficial capillary plexus; DCP, deep capillary plexus; CC, choriocapillaris.

One of the main characteristics of DR is retinal CNP, which can affect both the posterior pole and the periphery (6), with the latter being more frequently involved (7, 8). To this date, fluorescein angiography (FA) remains the gold standard for the identification of peripheral and macular CNP (2), and the advent of ultra-widefield (UWF) imaging has underlined its usefulness in the diagnosis and classification of DR (8). However, the requirement of an intravenous injection of dye, potentially leading to allergic reactions (9), and the two-dimensional visualization of the retinal vasculature, which does not allow to distinguish each vascular plexa (10), represent two of the main shortcomings of this technique. These limitations can be overcome with the use of optical coherence tomography angiography (OCTA), which is a safer, highly reproducible, non-invasive technique that allows a quantitative, three-dimensional assessment of the retinal circulation (Figure 2) (11). For these reasons, great effort in improving this technique has been made in the latest years, in particular to reduce the impact of its two greatest limitations: limited field of view and propensity to artifacts. Indeed, the latest commercially available UWF swept-source OCTA reaches a field of view up to 120° (12). Li et al. (12) recently demonstrated that when UWF swept-source OCTA is combined with UWF color fundus photograph, its detection rate of DR lesions is comparable [and even slightly superior for non-perfusion areas (NPAs)] to the classical combination of UWF color fundus photograph + UWF FA. Conversely, UWF OCTA is less capable of detecting microaneurysms in DR (12, 13). Nonetheless, the potential of this technique as an imaging modality in DR persists, particularly in terms of NPAs assessment, and may serve as a possible substitute for FA in the future (14).

## The role of capillary non-perfusion

### Peripheral capillary non-perfusion

Ultra-widefield FA has become the most popular imaging tool to visualize peripheral CNP, as it has been proved to display almost four times more non-perfusion than the traditional Early Treatment of Diabetic Retinopathy Study (ETDRS) seven-field images (15). Since peripheral CNP has been shown to be directly related to severity of the disease and the presence of predominantly peripheral lesions (8, 16, 17), the growing interest in its quantification is easily understandable. The most accepted method for quantifying NPAs in retinal vascular diseases is the ischemic index (ISI = NPA/retinal area) (7, 18, 19). Notably, CNP has been shown to correlate to a decreased fractal dimension, a surrogate measure of the complexity of vascular branching patterns, which can be useful when a precise computation of CNP is not possible (20).

With these tools, the role of peripheral CNP and its associations have been carefully dissected by several authors in the latest years. Results from the protocol AA, a 4 year prospective, longitudinal study that aimed to investigate the association of UWF FA findings with disease worsening in non-proliferative diabetic retinopathy (NPDR), found that greater baseline retinal CNP is associated with higher risk of DR progression (21).

In a recent study, the relation between DR severity and the extension of CNP has been investigated by means of UWF OCTA (11). In their study, Wang et al. (11) analyzed the correlation between the ratio of non-perfusion and the field of view of OCTA images and the severity of DR, with results comparable to those obtained with UWF FA by previous studies; moreover, they found that the peripheral region, especially the mid-periphery, is more prone to show DR lesions, as previously hypothesized by other authors (22).

Focusing on PDR, many attempts have been made to understand the influence of peripheral CNP on various DR biomarkers, such as VEGF levels and number and area of neovascular lesions (18, 23–25). Several authors found a correlation between the extent of NPAs and neovascular lesions area (25). In particular, larger areas of CNP seem to be associated with the presence of optic disc and posterior pole neovascularization (18, 23, 26). Additionally, a quantitative analysis by Ra et al. (24) unveiled that the extension of peripheral NPAs correlate with neovascular lesions area and is the strongest predictor of VEGF levels. Larger NPAs have also been correlated to increased level of inflammatory cytokines, suggesting inflammation as a possible target for suppressing non-perfusion-related PDR progression (27).

In a study conducted by Tong et al. (28) eyes with supra-large range non-perfusion, defined as the absence of capillaries beyond the posterior pole, were also at risk of ocular complications beyond just DR. In particular, eyes with supra-large range non-perfusion displayed higher incidences of neovascular glaucoma and diabetic keratopathy before surgery, greater need for intraocular tamponade during surgery, and higher probabilities of persistent corneal epithelial erosion and neovascular glaucoma recurrence after surgery (28).

Huang et al. (29) recently conducted a study focusing exclusively on patients with severe CNP. In their investigations they found that patients with extensively large areas of non-perfusion, defined as over 70% area of CNP throughout the whole image retina, are at high risk for rapid worsening of DR and unfavorable visual prognosis, and could benefit from more aggressive treatments (29).

On the contrary, a clear correlation between peripheral CNP and the presence of diabetic macular edema (DME) is still lacking. The DAVE study (7) investigated this topic and also addressed several issues that were present in previous UWF FA studies that aimed to understand the relation between peripheral CNP and DME, such as non-linear image distortion (30), the extent of the visible retinal area with non-montaged images (15) and the sub-classification of distinct retinal areas, on the basis of their different propensity for manifesting DR lesions (22). Results from this study did not find any association between the extent of CNP and the presence of DME, in accordance with a similar previous investigation (8); however, when considering the mid-periphery, CNP seemed to be negatively correlated with the presence of CME, which led the authors to hypothesize that CNP could be the result, rather than the cause, of VEGF production (7).

A novel approach developed by Jeong et al. (31) considered not only the NPAs, but also the spacial density of rod, cones and ganglion cells in the retina, to calculate the ratio of the cell number in the non-perfused retina to the cell number in the total retina, which they called “weighted ISI.” With this technique, they were able to find a correlation between weighted ISI, the level of various cytokines and DME, suggesting that the damage of more metabolically active regions plays a pivotal role in generating DME (31).

Finally, some authors also found that CNP can be used as a biomarker for many other alterations in patients with DR, such as white blood cell indexes (32) and renal function (33, 34).

### Diabetic macular ischemia

Diabetic macular ischemia (DMI) is another important complication of DR, often leading to visual loss in diabetic patients, which contrarily to DME is to this date irreversible (2). The first imaging modality used to recognize DMI was FA, which allowed the detection of an enlarged and irregular FAZ in patients affected by DR (35, 36). In the ETDRS, DMI on FA was determined by FAZ enlargement and outline disruption, and by capillary loss in the central subfield (37). The FAZ area also correlates with DR severity, increasing from NPDR to PDR (38). Finally, Sim et al. (39) found that FAZ enlargement, happening at a rate of 5−10% per year, also predicted progressive vision loss in eyes with overt DMI.

Even though FA proved to be useful in evaluating DMI, the impossibility to provide three-dimensional images and to assess the depth of the perfusion impairment represents an important drawback of this technique. Indeed, FA observations are mainly limited to the SCP, thus not allowing clear imaging of the deeper capillary networks. Conversely, OCTA allows the detection of SCP, DCP and ICP, making it possible to recognize different types of DMI (2). Differentiating between SCP and DCP has its relevance, since several studies have shown that DCP impairment correlates better than SCP with visual loss in DR (40–42). On the other hand, an impairment of both SCP and DCP results in a severe loss of visual function even in the absence of CME (43), and is often accompanied by structural changes of both the inner and outer retina, such as disorganization of the inner retinal layers (DRIL) and outer retinal atrophy (44). With OCTA, DMI can be distinguished on the basis of the predominant localization of ischemia (either the SCP or the DCP), with consequences on its effect on visual function and macular structure (2). In the predominant DCP-ischemia phenotype, there is a greater reduction in the vessel density (VD) in the DCP than the SCP (2), which seems to be a better predictor of DR severity and visual loss risk (45, 46). Moreover, non-perfusion in the DCP correlates with microstructural changes indicating photoreceptor damage, such as ellipsoid zone and external limiting membrane disruption and outer nuclear layer focal thinning (47). Recent studies involving adaptive optics-OCT unveiled severely altered morphology and density of cone photoreceptors in areas of CNP, together with a strong reduction of retinal sensitivity (48).

On the other hand, predominant SCP-ischemia is a phenotype characterized by a relative sparing of the DCP, and may represent a milder stage of the disease (2). This type of DMI preferentially involves the inner retina in the form of DRIL development and inner retinal thinning (49, 50). Tang et al. (51) found that a reduction of the SCP VD correlates with a thinner ganglion cell-inner plexiform layer (GC-IPL), suggesting a loss of ganglion cells. Likewise, VD of the SCP is lower in cases of DRIL, which is in turn associated with an enlargement of the FAZ (52). Nevertheless, Nicholson et al. (53) found that not all CNP areas are associated with DRIL, implicating that vascular impairment occur earlier than structural changes. Dodo et al. (54) recently demonstrated that NPAs in the SCP are associated to structural changes in the corresponding neuroglial components, displayed as regions with no boundary between the nerve fiber layer and the GC-IPL and spots with inverted OCT reflectivity. In the same work, the transverse length of NPAs in the DCP was associated with the length of cystoid spaces in the inner nuclear layer or Henle’s fiber layer (54).

Finally, DMI and DME seem to be strictly related with each other. Spaide (44) showed that a mismatch between the VD of the DCP and the SCP was associated with recurrent or persistent DME. Perhaps, the responsiveness of DME may be influenced by the degree of underlying DMI (55–57). In a study conducted by Murakami et al. (58) foveal cystoid spaces were associated with enlarged FAZ and microaneurysms in DME. Yalçın and Özdek (59) found that patients with more severe DME had a 1.04-fold greater chance of having macular ischemia on FA, and postulated that macular ischemia would become more likely as the cyst’s diameter grows. Furthermore, Mirshahi et al. (60) found that the presence of DME was associated with more extensive CNP when compared to DR eyes with no macular edema.

Despite its clinical relevance, there is still no consensus regarding the definition of DMI (61). Therefore, recent studies attempted to better characterize this entity, proposing more objective parameters for DMI definition, analyzing factors associated with its presence, and searching cut-offs to define vision-threatening DMI (61–63). Yang et al. (63) reported a series of associated factors of SCP-DMI and DCP-DMI, including older age, poorer visual acuity, thinner GC-IPL, worsened DR severity, higher hemoglobin A1c level, lower estimated glomerular filtration rate and higher low-density lipoprotein cholesterol level. Moreover, presence of DME and shorter axial length were associated with DCP-DMI. At the same time, Tsai et al. (62) investigated the correlation between microvascular parameters and visual function in eyes with DMI to identify threshold values to better define visual-threatening DMI. In a recent study, Terada et al. (61) demonstrated that the intercapillary spaces, a recently proposed objective method to quantify DMI, in the parafoveal and superficial vascular plexuses have significant impacts on visual acuity in DR without DME.

## Management of capillary non-perfusion

### Capillary non-perfusion in the absence of treatment

Several studies have considered the presence of CNP in eyes with DR and correlated it with various biomarkers, however, there remains a notable lack of knowledge regarding its natural history in DR, especially in the peripheral retina (25). Two studies have analyzed CNP longitudinal changes in untreated eyes.

The quantification of macular CNP through 2 years was performed in Reddy et al. (64) in a *post hoc* analysis of the phase 3 RIDE and RISE trials. These studies included eyes with DME, that had received no treatment during the preceding 3 months. Macular CNP was detected in 26.3% of sham eyes at baseline. On FA images, they estimated the percentage of capillary loss in disc areas at baseline, at 12 months and after 24 months, and observed a steady non-significant increase in macular CNP area among sham eyes at each time point.

In the AFFINITY trial (65), longitudinal variations of the non-perfusion index were tracked on UWF FA images over 1 year in 20 eyes with areas of CNP but without center-involved DME. They did not appreciate any rise of the non-perfusion index for their small sample at the end of the follow-up, emphasizing the need of studies with extended follow-up periods.

### Treatment of peripheral capillary non-perfusion

#### Continuous wavelength laser

The treatment options for peripheral CNP have broadened over the last decades. Historically, the effectiveness of laser therapy for managing DR has been proven in several major clinical trials (66, 67). Speculation surrounds the precise mechanism by which retinal laser therapy with continuous wavelength effectively treats and ameliorates retinal vascular disease. Regarding panretinal photocoagulation for PDR, one potential mechanism is that the damage induced by the treatment in poorly perfused areas reduces the retinal cell oxygen demand and the level of hypoxia, which results in a downregulation of angiogenic factors and VEGF production by the retinal tissue, as well as an increase in oxygen perfusion to the still-viable retina (68). Moreover, the diminished VEGF production also reduces vascular permeability and retinal edema.

The Diabetic Retinopathy Study, which evaluated the timing of panretinal photocoagulation in eyes with advanced NPDR and with PDR, was the first significant, prospective, multi-center, randomized clinical trial investigating the efficacy of retinal laser photocoagulation (67). In patients with high-risk PDR, this research showed that PRP was very effective and decreased the probability of severe vision loss by 60% at 2 years (67, 69).

However, conventional retinal photocoagulation has a number of serious potential side effects and disadvantages, including discomfort for the patient during the procedure, long treatment times (sometimes requiring multiple sessions), the potential for choroidal detachments after the procedure, increased intraocular pressure, CME, and decreased peripheral, color, and night vision for the patient (70, 71). Additionally, hemorrhage may occur as a direct result of treating retinal blood vessels or retinal neovascularization.

#### Anti-VEGF

Regarding the use of anti-VEGF agents, studies on peripheral ischemia have so far shown contradictory findings, which mostly depend on the imaging technique employed to measure it. Some authors found no appreciable peripheral CNP improvement with UWF FA in DR patients after anti-VEGF treatment over a follow-up of 3 to 12 months (72–74). A prospective UWF FA study comparing patients with PDR treated with intravitreal 2.0 mg aflibercept either monthly or quarterly revealed stability in the amount of CNP in patients receiving monthly aflibercept but not in those with lower dosage, leading the authors to hypothesize that the anti-VEGF agents dosage may affect the perfusion status (75). Other investigations showed either a decrease in the mean ISI on UWF FA or an improvement in retinal perfusion with promising outcomes in terms of peripheral ischemia in individuals with DR (25, 76–78). Similarly, Levin et al. (79) reported that 75% of eyes with DME and PDR treated with at least one intravitreal injection showed reperfusion of areas previously demonstrating CNP on UWF FA after 5 months.

On the other hand, in a prospective investigation conducted by Couturier et al. (73) despite a significant improvement in the DR severity scale score on color fundus photographs, none of NPAs present at baseline showed reperfusion in the arterioles, venules or capillaries after 3 monthly injections of anti-VEGF agents, on both UWF FA and UWF OCTA. According to their findings, anti-VEGF drugs did not have a protective effect on peripheral CNP.

### Dexamethasone implants

Querques et al. (80) evaluated early alterations in peripheral CNP following treatment for DME with a dexamethasone (DEX) implant on 9 eyes from 7 patients with NPDR. They assessed ISI at baseline and 10 weeks after a single intravitreal injection of DEX, which seemed to significantly improve retinal perfusion, with stability of the clinical picture after 1 year. They hypothesized that their outcome resulted from the favorable effects of corticosteroids on leukostasis, that had been implied in the development of DR for its effects vascular leakage and retinal non-perfusion (81, 82). However, these observations obtained from a small cohort of eyes have not found support by further studies. Hence, the low level of evidence cannot support the positive role of DEX implant on retinal perfusion status in DR.

### Treatment of macular non-perfusion

To this date, there are no treatment or prevention option available for DMI (2).

#### Anti-VEGF

It is widely known that VEGF-A plays a crucial role in both pathological and physiological angiogenesis as a growth and developmental factor. Furthermore, VEGF is expressed in retinal neurons, glia, and retinal pigment epithelium and is a survival factor for the retinal neurons and microvasculature (83, 84). It primarily binds to VEGFR2 to support endothelial migration and the integrity of the inner blood-retinal barrier. Therefore, the hypothesis that anti-VEGF drugs have a protective impact on DMI seems counter-intuitive. The current literature provides inconsistent information regarding the impact of anti-VEGF injections on macular CNP in patients with DR. Independent of the anti-VEGF molecule utilized, some studies have revealed no change in macular ischemia on FA after intravitreal injection in patients with DME and DR (77, 85) or with DME alone (86–89).

A retrospective *post hoc* analysis of the prospective RISE/RIDE studies, which included 666 DME patients treated with intravitreal ranibizumab or sham, revealed that despite all groups showed a rise in the percentage of patients with progressive posterior CNP from baseline to month 24, the progression was significantly quicker in the sham group at every time point between months 3 and 24, indicating that patients with DME may benefit from monthly anti-VEGF injections to delay the progression of retinal ischemia (64).

Conversely, in other studies involving either patients with (90–92) or without (93) DME, anti-VEGF therapy worsened macular CNP, with an expansion of the FAZ area seen on FA. In opposition with these findings, OCTA studies investigating the FAZ area and VD of the macular region in DME eyes with and without DR did not find any alteration of OCTA parameters following anti-VEGF treatment (78, 94–97). Noteworthy, a relatively short follow-up was a common drawback of most of these investigations.

A *post hoc* analysis of the RESTORE study revealed similar findings, without any significant change over 3 years of repeated ranibizumab injections in the FAZ area on OCTA (98). The possible danger of worsening macular perfusion in DR eyes has been pointed out by some authors who identified a considerable increase in the FAZ area, as well as a drop in VD following an anti-VEGF therapy course (86, 99). After three injections of 0.5 mg intravitreal ranibizumab, only one retrospective research with 50 DME eyes reported a substantial decrease in the FAZ region with a VD increase (100).

### Dexamethasone implants

Steroid medications are an effective treatment for DME, however, their effects on DMI are still unclear (101). It has been suggested that anti-VEGF may not be as effective as steroids on eyes with a predominance of inflammatory components. Eyes with more severe DMI might elicit a greater inflammatory response, which would then result in a worse outcome from anti-VEGF therapy alone. Additionally, the degree of visual recovery that can be attained may be constrained by the fact that DMI persists even after DME has subsided following anti-VEGF therapy (2).

#### Novel approaches

There are not any preventative measures or treatments available for retinal non-perfusion right now. To counteract ischemia, some proposed therapies focused on neurodegeneration, vasoregression, or pathological neovascularization (102). Lately, high dose systemic oxygen administration showed promising results in eyes with severe DMI (103). Another recently proposed target is the pathway of the semaphorins (63), which have a role in axonal growth cone guidance, immunological function, embryonic development, and adult circulatory vascular maintenance. In healthy adults, semaphorin levels are typically modest, while in diabetic patients they have been observed to be higher (104). Sema3a has been proposed to be a factor that specifically relates to regions of CNP and may be limiting revascularization of non-perfused tissues through its anti-angiogenic activities (5). Preclinical mouse models have shown that reduction of Sema3a increases rates of revascularization of avascular zones, which is consistent with its involvement in inhibiting revascularization of NPAs (5). Therefore, Sema3a is a possible target that might be inhibited in an effort to encourage revascularization of CNP regions. The lead compound of Sema Therapeutics, ST-102, is a bispecific recombinant trap protein that binds both VEGF-A and Sema3a and is currently undergoing pre-clinical testing for the treatment of DME.

Finally, Faricimab, a humanized, bispecific immunoglobulin G monoclonal antibody that binds and destroys Ang2 and VEGF-A has been used with good results to treat DME (105).

By competitively binding to Tie2, Ang2 adversely controls the Ang/Tie pathway by activating and destabilizing endothelial cells. The upregulation of molecules like ICAM-1 and VCAM-1 that results causes pericyte detachment, endothelial barrier breakdown, and an increase in the transmigration of macrophages and other inflammatory cells (106). The suggested Tie2 pathway-based vascular stabilization method may help DMI.

## Pathophysiological and therapeutical implications of central and peripheral capillary non-perfusion: Final remarks

In the present review, we provided an updated scenario regarding the characteristics, clinical impact, and management of central and peripheral CNP in DR (Table 1). CNP is an undoubtedly negative factor affecting diabetic retina, leading to the further progression of DR, to the possible onset of complications and to the overall worsening of retinal functionality. Indeed, besides the major morpho-functional changes such as DME or neovascularization, CNP has a functional negative impact on retinal sensitivity and visual function, representing a major cause of decreased quality of life of diabetic patients. To date, the current therapeutic strategies do not allow to properly manage CNP. Although some new therapies might represent meaningful steps forward for the treatment and improvement of central and peripheral CNP, the level of evidence is still too low to draw definite conclusions about this topic. Anti-VEGF treatments showed no effect on peripheral CNP (107) and DEX implants were not investigated in deep. Laser treatment is still a valuable option, especially for those patients showing low compliance to repeated intravitreal treatments. However, the impact on peripheral visual field should be carefully considered (108).

**TABLE 1:** Overview of peripheral and central capillary non-perfusion.

	Peripheral CNP	Central CNP
**Imaging**		
Most used tool	UWF FA	OCTA
Quantification Method	Ischemic index (ISI = NPA/retinal area)	Vessel density
**Clinical implications**		
Relation to DR Progression	Higher baseline CNP is associated with higher risk of DR progression	FAZ enlargement predicts progressive loss of visual acuity
Relation to PDR	Larger areas of CNP associated with optic disc and posterior pole neovascularization, neovascular lesion area and VEGF levels	FAZ area correlates with DR severity, enlarging from NPDR to PDR
Associated ocular complications	Higher risk of neovascular glaucoma and diabetic keratopathy	Irreversible visual acuity loss
Visual Prognosis	Extensively large areas of CNP indicate unfavorable prognosis	The involvement of both DCP and SCP can result in severe vision loss even in the absence of DME
Relation to DME	Contradictory findings	The degree of underlying DMI may influence the presence and responsiveness of DME, as well as the size of foveal cystoid spaces.
**Therapeutic options**		
Laser treatment	Panretinal photocoagulation lowers the probability of severe vision loss	No available options
Anti-VEGF	Contradictory findings	Faricimab may promote vascular stabilization
Dexamethasone Implant	Contradictory findings	Contradictory findings

CNP, capillary non-perfusion; UWF, ultra-widefield; FA, fluorescein angiography; OCTA, optical coherence tomography angiography; ISI, ischemic index; NPA, non-perfusion area; DR, diabetic retinopathy; PDR, proliferative diabetic retinopathy; NPDR, non-proliferative diabetic retinopathy; FAZ, foveal avascular zone; DME, diabetic macular edema; DMI, diabetic macular ischemia.

As outlined in the present review, multimodal retinal imaging provides a very powerful set of diagnostic approaches to assess the central and peripheral ischemic status of the retina in a non-invasive way. These examinations can be easily repeated, testing changes in the same retinal coordinates over time, thus providing an irreplaceable follow-up tool. Although quantitative multimodal retinal imaging has primarily been used for research purposes, its implementation in clinical settings could lead to a novel approach for patient classification and monitoring. This technology could enable precise categorization of patients based on their ischemic severity and allow for accurate monitoring of retinal perfusion changes during treatments. These techniques will help to better assess treatments efficacy and to customize therapeutic strategies. In addition, these approaches will provide new advances in knowledge regarding DR pathogenesis, contributing on the development of new molecules and technologies, as well as multitarget approaches to optimize DR management and to improve patients’ quality of vision and quality of life. Perhaps, the most interesting scenario would include the improvement of multi-target therapeutic approaches, focused on contrasting the pathologic cascades of mediators characterizing DR pathogenesis and favoring the reperfusion of those retinal regions affected by CNP.

As technical remark, it is important to consider that the current diagnostic approaches for evaluating central and peripheral CNP are prone to limitations. With respect to OCTA, the current technologies cannot allow to distinguish the real macular CNP from a displacement effect of macular capillaries secondary to DME; on the other side, when the macula is dry, the detection of real CNP is much more reliable (109). Looking at peripheral CNP, from one side the introduction of UWF technologies remarkably improved the amount of information achieved from the extreme periphery: on the other side, a consensus definition of peripheral CNP is still missed (109). Indeed, the previous definitions of 10-disc areas or 30-disc areas of CNP are less applicable in a so wide field of view. Moreover, the high curvature of the eye in the periphery reduces the intensity of the fluorescent signal, thus making less reliable the proper assessment of retinal perfusion in the extreme periphery. For all these reasons, further technological improvements are warranted to optimize the diagnostic workup of DR-related central and peripheral CNP.

## Author contributions

AAn and AAr: manuscript draft and conceptualization. LL, LB, EB, and EF: literature review and manuscript revision. FB and MB: expert revision of the draft. All authors have read and approved the final manuscript.
